# Circulating FGF21 Levels Are Progressively Increased from the Early to End Stages of Chronic Kidney Diseases and Are Associated with Renal Function in Chinese

**DOI:** 10.1371/journal.pone.0018398

**Published:** 2011-04-15

**Authors:** Zhuofeng Lin, Zhihong Zhou, Yanlong Liu, Qi Gong, Xinxin Yan, Jian Xiao, Xiaojie Wang, Shaoqiang Lin, Wenke Feng, Xiaokun Li

**Affiliations:** 1 School of Pharmacy, Wenzhou Medical College, Zhejiang, China; 2 School of Pharmacy, Jinan University, Guanghzou, China; 3 Division of Kidney, the 2nd Affiliated Hospital, Wenzhou Medical College, Zhejiang, China; 4 The Key Lab of Pathobiology, National Ministry of Education, Jilin University, Changchun, China; 5 School of Medicine, University of Louisville, Louisville, Kentucky, United States of America; University of Hong Kong, China

## Abstract

**Background:**

Fibroblast growth factor 21 (FGF21) is a hepatic hormone involved in the regulation of lipid and carbohydrate metabolism. This study aims to test the hypothesis that elevated FGF21 concentrations are associated with the change of renal function and the presence of left ventricular hypertrophy (LVH) in the different stages of chronic kidney disease (CKD) progression.

**Methodology/Principal Findings:**

240 subjects including 200 CKD patients (146 outpatients and 54 long-term hemodialytic patients) and 40 healthy control subjects were recruited. All CKD subjects underwent echocardiograms to assess left ventricular mass index. Plasma FGF21 levels and other clinical and biochemical parameters in all subjects were obtained based on standard clinical examination methods. Plasma FGF21 levels were significantly increased with the development of CKD from early- and end-stage (*P*<0.001 for trend), and significantly higher in CKD subjects than those in healthy subjects (*P*<0.001). Plasma FGF21 levels in CKD patients with LVH were higher than those in patients without LVH (*P* = 0.001). Furthermore, plasma FGF21 level correlated positively with creatinine, blood urea nitrogen (BUN), β2 microglobulin, systolic pressure, adiponectin, phosphate, proteinuria, CRP and triglyceride, but negatively with creatinine clearance rate (CCR), estimated glomerular filtrate rate (eGFR), HDL-c, LDL-c, albumin and LVH after adjusting for BMI, gender, age and the presence of diabetes mellitus. Multiple stepwise regression analyses indicated that FGF21 was independently associated with BUN, Phosphate, LVMI and β2 microglobulin (all *P<*0.05).

**Conclusion:**

Plasma FGF21 levels are significantly increased with the development of early- to end-stage CKD and are independently associated with renal function and adverse lipid profiles in Chinese population. Understanding whether increased FGF21 is associated with myocardial hypertrophy in CKD requires further study.

## Introduction

Chronic kidney disease (CKD) is a growing public health concern that is associated with a markedly increased risk of cardiovascular disease and mortality[1], [2]. Although many traditional risk factors for atherosclerosis such as hypertension and diabetes mellitus promote the progression of CKD from early- to end-stage, these classic risk factors do not fully account for the burden of cardiovascular disease in patients with CKD[3], [4]. Left ventricular hypertrophy (LVH) is one of several common manifestations of cardiovascular disease that is a major independent risk factor for mortality in patients with CKD[3], [4]. Exploring the early mechanism of LVH is necessary to develop therapies that block the progression of CKD and attenuate cardiovascular disease associated with CKD.

The fibroblast growth factor family is composed of 22 members with a wide range of biological functions, including cell growth, development, angiogenesis, and wound healing[5]–[9]. FGF21 is a member of the endocrine FGF subfamily, which also includes FGF23, human FGF19, and its mouse homolog FGF15[10]–[14]. In mice, FGF21 is expressed predominantly in the liver and stimulates glucose uptake through the induction of GLUT1 in adipocytes[13]. In vivo treatment with FGF21 results in amelioration of glucose and regulates lipid metabolism in both murine and nonhuman primate models of diabetes and obesity[15]–[17]. Taken together, these findings demonstrate an important role of FGF21 as a hepatic hormone in the regulation of lipid metabolism and also suggest that FGF21exhibits the therapeutic characteristic necessary for an effective treatment of obesity and fatty liver disease.

Human studies indicate that circulating levels of FGF21 increased in obese individuals [18], subjects with metabolic syndrome, type 2 diabetes mellitus[19]–[21] and coronary heart disease[22]. Furthermore, FGF21 was found to closely associated with renal dysfunction in end-stage renal disease subjects[23], [24]. On the other hand, previous studies indicated that FGF receptors, particularly FGFR1, are expressed in adult myocardial cells, and their activation by locally secreted growth factors can stimulate myocardial hypertrophy and interstitial fibrosis[25], [26]. For example, mice lacking FGF-2 exhibit dilated cardiomyopathy and impaired hypertrophic responses to angiotensin II[27], and adenoviral Gene Transfer of FGF-5 to Hibernating myocardium improves function and stimulates myocytes to hypertrophy and reenter the cell cycle[26]. Recently, FGF-23, one member of FGF-19 subfamilies, was reported to associate with the onset and development of CKD[28]–[30] as well as LVH in patients with CKD[31]. Take together, all these reports suggested that FGF family members play an important role in the physiopathology of LVH. Whether FGF21, however, is closely associated with the pathological relevance of CKD and LVH in CKD patients remain unclear. To explore the physiological and pathological relevance of FGF21 in patients with CKD, we measured the plasma concentrations of FGF21 in 240 Chinese subjects and analyzed its association with renal function and a cluster of metabolic parameters that related to the change of renal functions.

## Materials and Methods

### Study Population

The study population consisted of 146 outpatients with CKD and 54 CKD patients with long-term hemodialysis. 146 patients with CKD were recruited from outpatient nephrology clinics at the Second Affiliated Hospital of Wenzhou Medical College, Wenzhou. Patients recruited into this study were ≥25 years of age and had a sustained reduction (≥3 months) in estimated glomerular filtration rate (eGFR) ≤60 ml · min^−1^ · 1.73 m^-2^ based on the simplified Modification of Diet in Renal Disease formula. Patients with preserved eGFR (≥60 ml/min per 1.73 m^2^) were also considered as early stage CKD patients if they presented with one or more of the following related symptoms: persistent hematuria and/or proteinuria with biopsy-proven minimal-change disease, membranoproliferative glomerulonephritis, or other relevant nephropathies. 54 CKD patients with long-term hemodialysis (eGFR<15 ml · min^−1^ · 1.73 m^−2^) were also recruited into the present study. These subjects with long-term hemodialysis were recruited from inpatient services at the Hemodialytic Center of the 2^nd^ Affiliated Hospital of Wenzhou Medical College and were scheduled for an echocardiogram for diagnostic purposes by the primary admitting team. Patients were at least 25 years of age and clinically stable and did not have acute myocardial infarction, known cardiomyopathy or ejection fraction < 40%, or known mitral or aortic valve disease. Exclusion criteria included kidney transplant, history of coronary artery bypass grafting, or an incidence of myocardial infarction within 90 days of enrollment. In addition, to focus on patients with early cardiac disease, patients surpassing criteria for New York Heart Association class 1 heart failure or Canadian Cardiovascular Society class 1 angina were excluded. The comorbidities including diabetes and hypertension were defined on basis of the clinical diagnosis criteria of WHO and American Diabetes Association or American Society of Hypertension respectively. We also included healthy controls (n = 40) who underwent a routine health examination at 2nd Affiliated Hospital of Wenzhou Medical College, had no history of medical disease, and were not taking regular medication, all control subjects were selected based on the results of physician's questionnaire and clinical biochemical examination. All studies were approved by the Ethics Committee of Wenzhou Medical College, and all patients provided written informed consent.

### Clinical Data and Laboratory test

Data on demographic characteristics, medical history, current medications, and blood samples were collected from all subjects at the time of enrollment. Blood was collected from subjects after overnight fasting for at least 10 h. Blood samples were immediately centrifuged, separated into aliquots, and stored at −80°C for future batched assays. Serum creatinine, calcium, phosphate, and albumin were measured with standard commercial assays. Intact parathyroid hormone concentrations were measured with the Roche Elecsys parathyroid hormone assay (Roche, Indianapolis, Ind). Plasma FGF21 (Biovendor, Modrice, Czech Republic) and C-reactive protein (CRP, R&D, USA)concentrations and were measured in duplicate with commercially available enzyme-linked immunosorbent assays according to the manufacturers' instructions in the Core Laboratory of School of Pharmacy, Wenzhou Medical College. All other blood tests in CKD subjects were processed in Clinical Examination Laboratory of the 2^nd^ Affiliated Hospital of Wenzhou Medial College after a single thaw.

### Echocardiography

All subjects underwent 2-dimensional transthoracic echocardiograms. The studies were interpreted by a single reviewer at the 2^nd^ Affiliated Hospital of Wenzhou Medial College who was blinded to subjects' clinical and laboratory data. For the primary analysis, left ventricular mass index (LVMI) was calculated with the Devereux formulation[32], [33]: Left ventricular hypertension (LVH) was defined as LVMI >135 g/m^2^ for men and >110 g/m^2^ for women in Chinese population[34]. Left ventricular ejection fraction (LVEF) was determined using biplane-modified Simpson's measurements.

### Statistical analysis

All analyses were performed with Statistical Package for Social Sciences version 13.0 (SPSS,Chicago, IL), and the statistical analysis was done similarly as described by Zhang X et al[18]. In brief, normally distributed data were expressed as mean±SD. Data that were not normally distributed, as determined using Kolmogorox-Smirnov test, were logarithmically transformed before analysis and expressed as median with interquartile range. Student's unpaired t test was used for comparison between two groups. Pearson's correlations were used as appropriate for comparisons between groups, and multiple testing was corrected using Bonferroni correction. Linear regression was used to examine the association between LVMI, LVH, CRP and other clinical, and laboratory variables. We used multivariable models to examine the relationship between LVMI and FGF21 concentrations, adjusting for age, gender, body mass index (BMI), diabetes mellitus, hypertension that were significantly (*P*<0.05) associated with LVMI in univariate analyses. The variables that correlated significantly with serum FGF21 (after Bonferroni correction for multiple testing) were selected to enter into multiple linear regression. In all statistical tests, P values <0.05 were considered significant.

## Results

### Patient Characteristics

Table 1 depicts demographic information, laboratory data and echocardiogram results of the 200 CKD subjects according to level of kidney function and 40 normal control subjects. CKD patients were divided into three groups according to the eGFR, namely early-stage group (preserved renal function, eGFR 60 to 90 ml/min per 1.73 m^2^), middle-stage group (eGFR 30 to 60 ml/min per 1.73 m^2^) and end-stage group (hemodialytic group, eGFR <30 ml/min per 1.73 m^2^). 55 patients with preserved eGFR (>90 ml/min per 1.73 m^2^) were also considered as CKD patients (early stage) based on the recruitment standards shown in the Materials and Methods section. Furthermore, relevant biochemical and anthropometric parameters in 40 healthy subjects were shown in Table 1. The results of echocardiography and its relevant parameters in normal subjects were not examined in this study, because all these subjects recruited into the present study were underwent a routine health examination, and had no history of disease, and were not taking regular medication based on the results of physician's questionnaire and clinical biochemical examination.

**Table 1:** Description of Subjects by Level of Kidney Function.

Variables	Normal subjects	Early-stage eGFR >60ml/min per 1.73 m^2^	Middle-stage eGFR(30–60)ml/min per 1.73 m^2^	End-stage eGFR <30ml/min per 1.73 m^2^	*P* for trend
N	40	76	36	88	-
Age(years)	49.5±12.3	50.7±15.0	50.7±17.3	50.6±10.0	NS
Women(%)	14(32.5)	25(32.8)	12(33.3)	28(31.8)	NS
BMI(kg/m^2^)	21±2.5	23±3.3	23.8±3.6	21±2.9	<0.001
Hypertension					
Systolic blood press	104±18	136±24	149±28	150±31	<0.001
Diastolic blood press	69±11	84±17	88±14	85±19	<0.001
The presence of hypertension	-	36.8	55.6	50.0	NS
Glucose					
fasting glucose	5.08±0.56	4.99±1.37	4.59±1.02	7.59±4.52	<0.001
fasting insulin	4.16(3.01–6.17)	7.62(4.96–11.37)	13.82(8.06–30.95)	12.99(5.08–23.16)	<0.001
HOMA-ir	0.92(0.63–1.44)	1.31(0.77–2.27)	4.63(2.47–5.34)	3.58(1.04–7.26)	<0.001
The presence of diabetes	-	19.7	33.3	46.6*	<0.001
Lipid profiles					
TG	0.92±0.38	2.05±1.20	1.97±1.37	1.93±1.49	0.004
LDL	2.57±0.46	3.30±1.60	3.30±1.23	2.26±0.46	<0.001
HDL	1.39±0.25	1.15±0.50	1.09±0.40	0.94±0.31	<0.001
Laboratory values					
CCR	81.2±13.5	66.2±27.5	28.2±10.7	7.5±3.5	<0.001
Albumin(g/l)	48.7±6.7	30.4±8.8	28.8±7. 4	30.9±4.6	<0.001
Creatinine(µmol/l)	65.8±11.0	103.9±39.3	243.6±47.5	811.3±293.3	<0.001
Calcium (mmol/l)	2.28±0.32	2.13±0.17	2.16±0.12	2.19±0.21	NS
phosphate(mmol/l)	1.24±0.26	1.26±0.22	1.36±0.16	1.78±0.61	<0.001
PTH(pg/ml)	42.3±17.0	76.7±26.0	97.6±64.7	469.0±470.0	<0.001
BUN(mmol/l)	4.1±1.04	6.35±2.67	12.28±4.58	21.44±7.51	<0.001
Uric Acid(µmol/l)	286±51	388±98	431±118	443±120	<0.001
CRP(µg/ml)	0.18±0.16	0.71±0.53	1.17±1.29	2.02±1.68	<0.001
β2 microglobulin(µg/ml)	1.51±0.32	2.62±1.09	5.90±1.24	15.50±9.87	<0.001
Adiponectin(µg/ml)	2.10±0.78	2.26±0.65	2.58±0.59	3.26±1.17	<0.001
FGF21(pg/ml)	127.6(85.7 to 218.4)	317.1(209.9 to 732.8)	517.1(220.3 to 912.0)	1098.8(523.1 to 2467.8)	<0.001
Echocardiography					
LVMI(g/m^2^)	-^#^	100.6±21.7	112.3±14.7	136.6±39.7	<0.001
LVH,%	-^#^	12.0	28.6	53.7	<0.001
EF,%	-^#^	66.7±3.5	64.4±6.5	62.8±5.9	<0.001

NS: not significant. Results are expressed as frequencies, mean±SD, or median (interquartile range) as appropriate.

*Percentage of type 2 diabetes mellitus in end-stage CKD patients is significantly higher than those CKD patients in early-and middle-stage.

### Plasma FGF21 levels are increased in CKD patients

**Figure 1:**
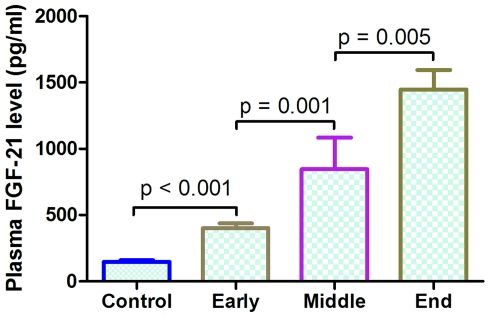
Plasma FGF21concentration in different stage of CKD subjects and normal subjects.

Plasma FGF21 levels ranged from 67.4 to 7232.6 pg/ml amongst subjects in this study, and the median FGF21 concentration in overall subjects was 616.1 pg/ml (interquartile range, 264.4 to 1137.8 pg/ml). No gender differences in plasma FGF21 levels were found between men (826.3±100.4 pg/ml, n = 155) and women (789.6±86.5 pg/ml, n = 85; p = 0.470). After adjusting for age, gender, and body mass index (BMI), median circulating FGF21 level in end-stage CKD patients (1447.9±146.5 pg/ml) was 10-fold higher compared with normal subjects (147.3±12.25 pg/ml; *P*<0.0001); and about 3.5-fold higher compared with the early-stage CKD patients (402.9±35.0 pg/ml; *P*<0.0001), and about 1.5-fold higher compared with the middle-stage CKD patients (845.8±238.7 pg/ml; *P*<0.0001; Figure 1). Interestingly, CKD patients recruited into this study with diabetes mellitus (DM) (1253±170.1 pg/ml, n = 68) had significantly higher plasma FGF21 levels (*P* = 0.0016) than those without DM (714±82.1 pg/ml, n = 132). On the other hand, no significant difference in plasma FGF21 levels was observed between all CKD patients with and without hypertension (*P* = 0.7730), as well with and without CHD (*P* = 0.6563). Furthermore, all CKD subjects with other comorbidities including DM, hypertension and CHD in this study had significantly higher plasma FGF21 levels (1422±302.9 pg/ml, n = 9) than those without corresponding comorbidities (611.2±111.5 pg/ml, n = 55, p = 0.0091).

### FGF21 and left ventricular structure

LVMI values in all CKD subjects of this study ranged from 49.02 to 247.99 g/m^2^ with mean LVMI at 118.5 g/m^2^ and 31.7% of patients experiencing left ventricular hypertrophy. Compared with early- and middle-stage CKD patients, however, patients with end-stage CKD had significantly higher mean LVMI(110.3 ± 28.7 *vs.* 132.8 ± 36.1 g/m^2^) and lower mean ejection fraction(67.97±3.40 *vs.* 63.33±4.97%). Mean LVMI and the prevalence of LVH (31.7%) among CKD subjects in this study were lower than in previous studies[35], [36] confirming that the majority of patients recruited for this study were free of significant cardiac disease, consistent with the study design. Furthermore, CKD patients with hypertension showed a higher LVMI value than those without hypertension (124.9±34.8 *vs.*110.9±32.8, *P* = 0.006).

**Figure 2:**
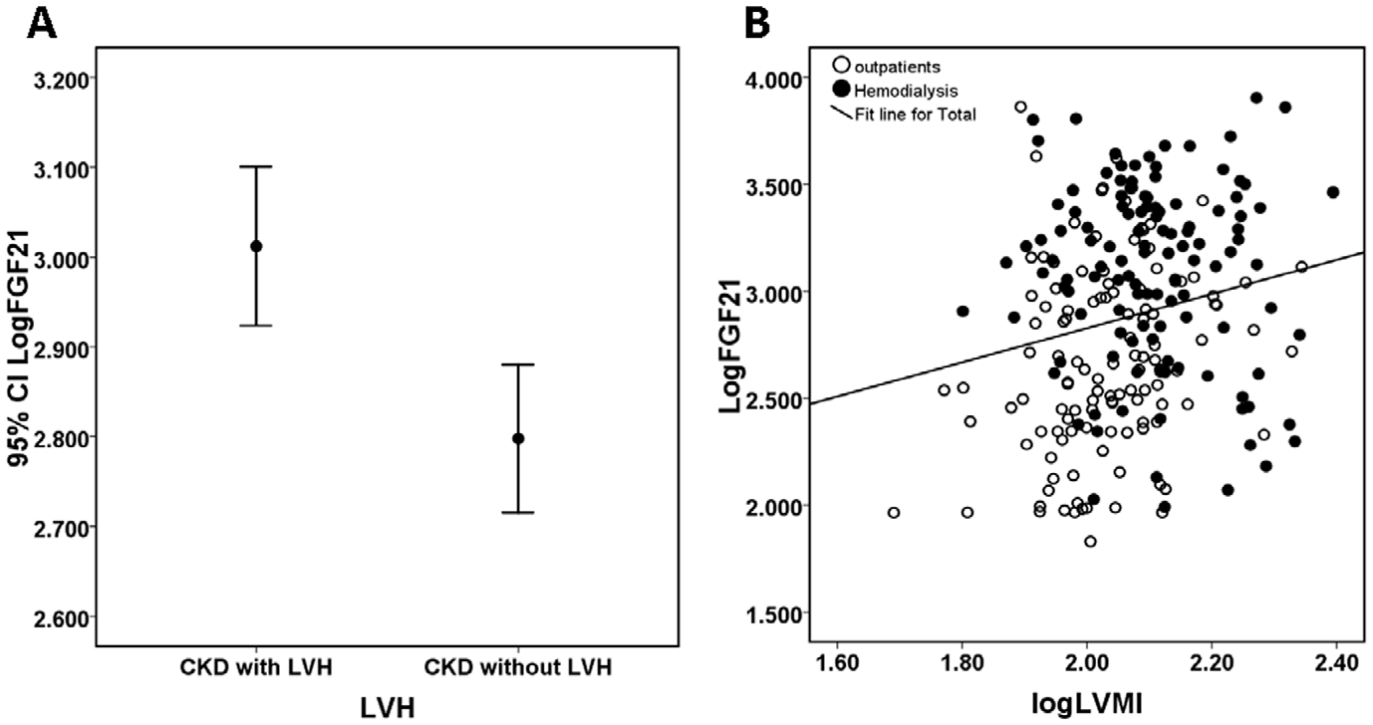
The relationship between FGF21 and LVH. A, circulating levels of FGF21 in CKD patients with LVH were significantly higher than that of CKD subjects without LVH; B, correlation between log-transformed FGF21 and LVMI. ○ indicates outpatients (early-and middle-stage CKD patients); • hemodialytic patients (end-stage CKD patients).

To explore the relationship between FGF21 and ventricular structure, we compared the levels of FGF21 in the CKD patients with and without LVH. Plasma FGF21 levels in CKD patients with LVH (median 923.9 pg/ml, interquartile 363.7 to 1478.7 pg/ml) were significantly higher than those without LVH (median 446.5 pg/ml, interquartile 245.6 to 953.1 pg/ml; *P* = 0.001; Figure 2A).

In univariable analyses, increased log FGF21 concentrations were significantly associated with increased LVMI (*P* = 0.007; Figure 2B). When adjusted for age, gender, BMI, diabetes mellitus, no significant association was found between log FGF21and log LVMI (*P* = 0.091). In the multivariable model, BMI, systolic pressure and LVH were the only three parameters that remained significantly associated with LVMI (*P*≤0.003 respectively). Furthermore, when examined in tertiles, mean LVMI increased with increasing tertiles of log FGF21(*P* = 0.005 for trend; Table 2).

**Table 2:** Echocardiographic characteristics and relevant interesting factors by tertile of log FGF21.

variable	Log FGF21(< 2.5) Tertile 1	Log FGF21 (2.5 to 3.0) Tertile 2	Log FGF21 (>3.0) Tertile 3	*P* for trend
**N**	52	51	82	-
**LVMI,(g/m^2^)**	109±32	119±31	127±35	0.004
**LVH, %**	21	34	46	0.006
**Phosphate(mmol/l)**	1.40±0.42	1.46±0.45	1.65±0.63	0.007
**CRP(µg/ml)**	0.62(0.22–1.28)	0.89(0.82–1.82)	1.47(0.71–3.45)	< 0.001

Results are expressed as mean±SD, frequencies (%) and median (interquartile) as appropriate.

On the other hand, in univariable analyses, increasing log FGF21 concentration was also significantly associated with the presence of LVH (odd ratio[OR] per 1-SD increase in log FGF21, 0.381; 95% confidence interval [CI], 0.214 to 0.68; *P* = 0.001). When adjusted for age, gender, BMI, hypertension, diabetes mellitus, log FGF21 was still significantly associated with the presence of LVH (OR per 1-SD increase in log FGF21, 0.422; 95% CI, 0.218 to 0.817; *P*  = 0.010). Furthermore, the prevalence of LVH increased significantly with ascending tertiles of log FGF21 in univariable analyses (Table 2), but this relationship was attenuated after multivariable adjustment.

### FGF21 and Phosphate Concentrations

**Figure 3:**
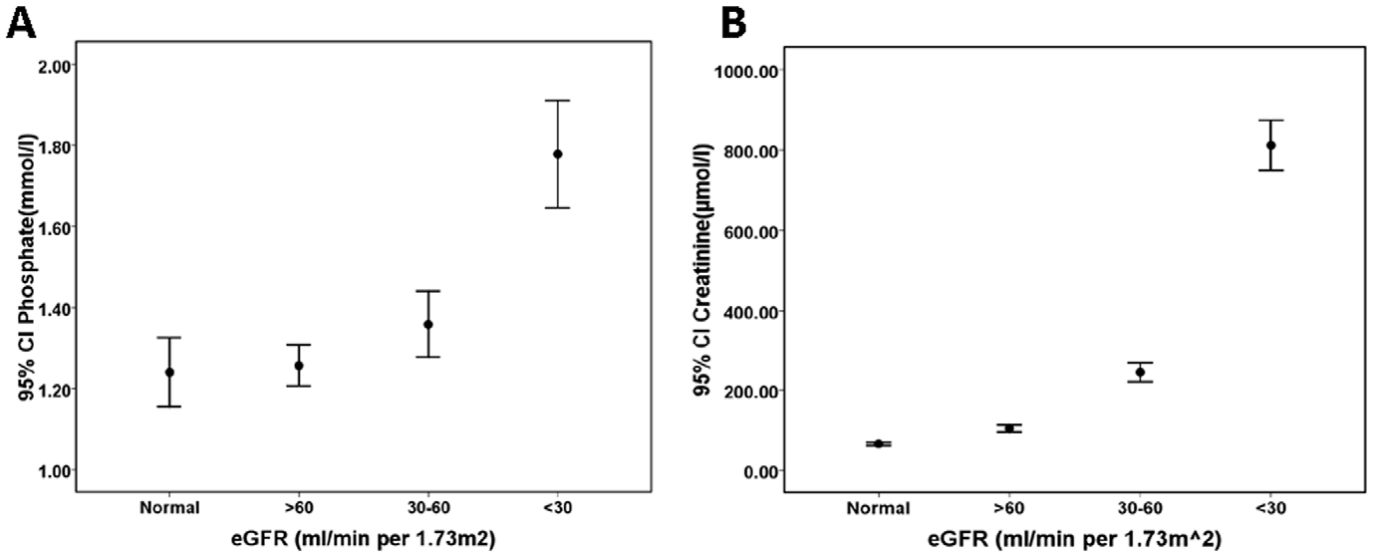
The change tendency of serum phosphate and creatinine according to the level of eGFR in 240 Chinese subjects. Bars represent SDs.

Hyperphosphatemia with serious inflammatory reaction is a common manifestation of CKD. As shown in Table 3, circulating levels of FGF21 were found to strongly correlate with serum phosphate, creatine and CRP levels in CKD subjects after adjustment for BMI, gender, age and diabetes mellitus. Furthermore, mean FGF21, phosphate and creatinine concentrations were significantly increased with decreasing levels of eGFR(P for trend <0.001 for both). However, the absolute difference in mean serum phosphate concentration between normal subjects and early-stage CKD patients was about 0.01 mmol/l (relative difference, increasing 0.8%, *P* = 0.727; Figure 3A); Furthermore, the absolute difference in mean serum creatinine levels between normal subjects and early-stage CKD patients was about 38.1 µmol/l (relative difference, increasing 57.8%, *P*<0.001; Figure 3B). In contrast, the absolute difference in mean FGF21 concentration between normal subjects and early-stage CKD group was 245 pg/ml (relative difference, increasing 1.5-fold, *P*<0.001). Additionally, a significant difference in FGF21 concentration was found between early-and middle-stage CKD patients (402.4±49.6 *vs.* 904.6±262.0 pg/ml; *P* = 0.0014; Figure 1A).

**Table 3:** Correlations of serum FGF21 levels with anthropometric parameters, biochemical indexes as well as other relevant factors.

Variables	Plasma FGF21 #		Plasma FGF21 # *	
	r	p	r	p
Gender	-0.201	0.004	-	-
BMI	-0.158	0.026	-	-
Age	0.186	0.035		
Diabetes	-0.310	<0.001	-	-
Systolic pressure	0.255	<0.001	0.243	0.001
CCR#	-0.503	<0.001	-0.349	<0.001
eGFR#	-0.469	<0.001	-0.341	<0.001
BUN#	0.413	<0.001	0.306	<0.001
Creatinine#	0.513	<0.001	0.412	<0.001
CRP	0.271	<0.001	0.178	0.013
Adiponectin	0.221	0.008	0.220	0.009
CXCL16	0.24	0.001	0.134	NS
TG	0.147	0.013	0.153	0.015
Cholesterol	-0.151	0.011	-0.095	NS
HDL-c	-0.250	<0.001	-0.189	0.003
LDL-c	-0.250	<0.001	-0.158	0.012
Proteinuria	0.318	0.001	0.302	0.001
Fasting glucose	0.263	<0.001	0.165	0.009
Albumin	-0.165	0.019	-0.145	0.047
β2 microglobulin	0.850	<0.001	0.930	<0.001
Phosphate	0.288	<0.001	0.261	<0.001
LVDd	0.194	0.024	0.171	NS
LVMI	0.221	0.008	0.113	NS
LVH	-0.217	0.001	-0.174	0.009

# Log transformed before analysis;

*adjusted by age, gender, BMI and diabetes; NS, not significant; CCR: endogenous creatinine clearance rate; IVSTd, left ventricular end-systolic dimension; LVDd: left ventricular diastolic dimension; LVMI: left ventricular mass index; LVH: left ventricular hypertrophy.

### Plasma FGF21 levels strongly associated with renal function, adverse lipid profiles and other factors in all CKD subjects

**Figure 4:**
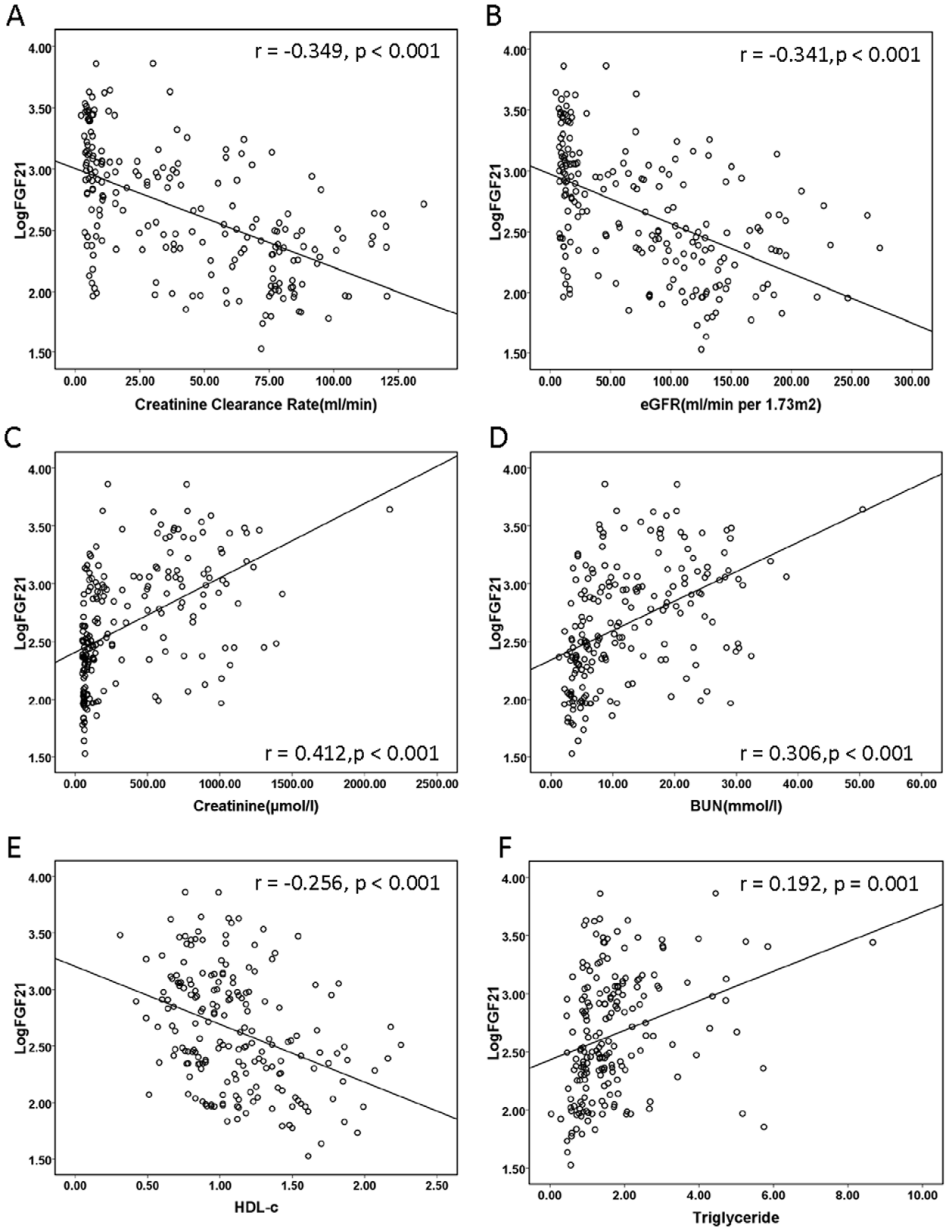
Correlation of plasma level of FGF21(log transformed) with CCR(A), eGFR(B), Creatinine(C), BUN(D), HDL-c(E) and Triglyceride(F) after adjustment for age, gender, BMI and diabetes in 240 Chinese subjects.

As shown in Table 3 and Figure 4, plasma FGF21 levels were strongly correlated with eGFR, endogenous creatinine clearance rate (CCR), creatinine and BUN, suggesting that elevated FGF21 concentrations were closely associated with the renal function in these CKD subjects. On the other hand, plasma FGF21 levels were associated with adverse lipid profiles including HDL-c, LDL-c and triglyceride (all P<0.05) after adjustment for age, gender, BMI and diabetes mellitus in all CKD subjects. Furthermore, systolic pressure, adiponectin, proteinuria, β2 microglobulin, phosphate, albumin and LVH were significantly correlated with circulating FGF21 levels after adjustment for BMI, gender age and diabetes mellitus in all CKD subjects.

### Independent Association of Plasma FGF21 levels with β2 microglobulin, LVMI, and Diabetes Mellitus

To determine whether plasma FGF21 was independently associated with anthropometric parameters and other relevant factors, multiple stepwise regression analysis involving all the parameters with significant correlations with plasma FGF21 was performed. Multiple stepwise regression analysis revealed that plasma FGF21 was independently associated with BUN, Phosphate, LVMI and β2 microglobulin adjustment for age, gender and BMI in all CKD subjects (*P*≤0.043, respectively, Table 4), thus all other parameters including gender, BMI, diabetes, systolic pressure, CCR, eGFR, creatinine, BUN, CRP, adiponectin, CXCL16, triglyceride, total cholesterol, LDL, HDL, proteinuria, fasting glucose, albumin, LVDd and LVH were excluded during regression analysis.

**Table 4:** Multiple stepwise regression analysis showing variables independently associated with the plasma level of FGF21.

Independent variables	Standardized Coefficients (Beta)	t	Sig.	B (95% CI)
**BUN**	1.066	3.406	0.004	0.046(0.017 to 0.075)
**LVMI**	-0.426	-2.676	0.017	-0.007(-0.012 to -0.001)
**Phosphates**	-0.992	-3.229	0.006	-0.747(-1.240 to -0.254)
**β2 microglobulin**	0.631	2.209	0.043	4.194E-5(0.000 to 0.000)

The analysis also included gender, BMI, diabetes, systolic pressure, CCR, eGFR, creatinine, BUN, CRP, adiponectin, CXCL16, triglyceride, total cholesterol, LDL, HDL, proteinuria, fasting glucose, albumin, LVDd and LVH, which were excluded during regression analysis.

## Discussion

Although several clinical studies focusing on FGF21 and its relevant human diseases have been reported in recent years[18]–[24], the definitive mechanism of FGF21 is not fully understood. In the present study, we investigated (1) the clinical correlation of circulating FGF21 in conjunction with physiological and pathological aspects in CKD patients, (2) the relationship between the change of circulation FGF21 levels and the development of CKD from early- to end-stage of disease, and (3) the effect of comorbidities of CKD to plasma FGF21 concentration. Our results suggest that FGF21 is involved in the development of CKD as supported by two novel findings. First, plasma FGF21 concentration is increased with the development of CKD from early- to end-stage following the loss of renal function. Second, relevant comorbidities including DM, hypertension and CHD in CKD patients affected plasma FGF21 concentration. Furthermore, plasma FGF21 levels in CKD patients are significantly correlated with adverse lipid profiles and LVH, but not with LVMI after adjustment for age, gender, BMI and diabetes.

Previous studies indicated that serum FGF21 concentration was 15-fold higher in chronic hemodialytic patients (CKD end-stage) than normal subjects and was associated significantly with the loss of renal function[24]. More recently, a cross-sectional study also indicated that serum FGF21 concentration was associated with residual renal function and insulin resistance in end-stage CKD patients with long-term hemodialysis[23]. These results suggested that FGF21 may be related to renal excretion functions in humans. Consistent with these reports, we show that plasma FGF21 concentration is increased with the development of early- to end-stage CKD following the loss of renal functions in CKD patients. Our data indicated that plasma FGF21 levels in end-stage CKD patients are about 10-fold higher compared with normal subjects (Figure 1). Furthermore, plasma FGF21 levels in end-stage CKD patients are about 4.5-fold higher compared with the early-stage CKD patients; and about 1.5-fold higher compared with the middle-stage CKD patients (Figure 1). These results suggest that circulating FGF21 concentration is associated with the CKD progression.

Diabetes contributes to increased morbidity and mortality in patients with chronic kidney disease[37]. Diabetes is the single leading cause of kidney failure in the U.S., accounting for about 45% of people who start treatment for kidney failure each year[38]. In the present study, we found that diabetes has additional effects on FGF21 levels in CKD patients, which are supported by the fact that plasma FGF21 levels in CKD individuals with DM were significantly higher than those CKD patients without this complication (Figure 1B). Furthermore, stepwise logistic regression analysis revealed that plasma FGF21 was independently associated with diabetes. Take together, these results suggest that plasma FGF21 levels in CKD subjects are affected by relevant comorbidities s and as on.

Hyperphosphatemia with serious inflammatory reaction is a common manifestation of CKD, and has been shown to be significantly associated with FGF-23, one of members of FGF family[28], [39]. In the present study, we found that elevated FGF21 levels were associated with increasing serum phosphate and CRP levels. The relative difference in mean serum phosphate concentration between normal subjects and early-stage CKD patients was significantly lower than that of plasma FGF21 levels (relative difference, 0.8% *vs.*150%). These results suggest that FGF21 is a better biomarker than blood phosphate to reflect the progression of CKD from the early-to middle stage.

Left ventricular hypertrophy (LVH) is one of several common manifestations of cardiovascular disease and is an independent risk factor for mortality in patients with CKD[3], [4]. Previous studies showed that approximately 40% of patients with pre-dialysis CKD and up to 80% of patients initiating hemodialysis manifest LVH[4], [36]. Furthermore, FGF-23, one member of the FGF-19 subfamilies, was shown to associated with LVH in patients with CKD[31]. In the present study, the prevalence of LVH (39.7%) among all CKD subjects was lower than in previous studies, because the majority of patients recruited for this study were free of significant cardiac disease. Furthermore, our data indicated that CKD patients with hypertension have a higher LVMI level than those of patients without hypertension, suggesting hypertension lead to an increase in the percentage of LVH. However, no significant difference in plasma FGF21 level was found between CKD patients with and without hypertension. These results indicated that hypertension can lead to increase LVMI levels but do not affect circulating levels of FGF21 in CKD patients.

Previous clinical studies indicated that the plasma lipid profile frequently evolves during the course of progression of CKD, and dyslipidemia is a strong predictor of myocardial infarction in sujects with CKD[40], [41]. Patients with mild to moderate CKD, especially those with significant proteinuria, commonly exhibit hypercholesterolemia and elevated LDL levels[42]. Serum triglycerides and very low-density lipoprotein (VLDL) levels are elevated, and clearance of VLDL and chylomicrons and their atherogenic remnants is impaired in patients with advanced CKD or end stage renal disease (ESRD)[42], [43]. In present study, our data showed that elevation of FGF21 are significantly correlated with adverse lipid profiles including elevating LDL and triglyceride, as well as decreasing HDL after adjustment for age, gender, BMI and diabetes suggesting that the elevation of circulating FGF21 levels may be directly or indirectly linked to the progression of pathophysiology of CKD.

eGFR, CCR, creatinine and BUN are conventional biomarkers reflecting the change of renal function[2], [30]. Recent studies indicated that serum levels of cystatin C and β2 microglobulin (β2 MG) as well as urinary β2 MG and N-acetyl-β-Dglucosaminidase (NAG) increase in patients with early and mild renal impairment[44]–[47]. In the present study, our data indicated that plasma FGF21 levels are independently associated with β2 microglobulin, and were significantly associated with eGFR, CCR, creatinine and BUN in CKD subjects(Figure 4), suggesting that elevated FGF21 levels are closely related to the injury of glamorous and the change of renal function in CKD patients. However, the mechanisms responsible for the elevation of FGF21 concentration with the progression of CKD are not fully understood. FGF21 is expressed predominantly in liver and adipose tissue[13], and plays an important role in regulating lipid and energy metabolism[15], [48]. We speculated that the paradoxical increase in CKD patients is a compensatory mechanism to counteract metabolic stress. Because FGF21 resistance might be found in obesity and in renal failure, leading to compensatory upregulation of this hepatokine[18], [24]. Based on these findings, we propose that the mechanism of increased FGF21 levels in kidney disease is similar to those observed in obesity-associated resistance to insulin. Further studies are needed to elucidate the precise mechanism by which CKD subjects elevate circulating FGF21 levels and to reveal the role of increased FGF21 levels in the onset and development of CKD.

In summary, this study provides clinical evidence revealing that plasma concentrations of FGF21 are increased with CKD progression and are independently associated with the loss of renal function. There are several limitations in this study. The sample size of this study cohort is relatively small. Furthermore, the cross-sectional natures of this study do not allow us to address the causal relationship between FGF21 and the development of CKD in patients. Further prospective studies with larger sample sizes are needed to determine whether FGF21 is related to myocardial hypertrophy in patients with CKD.
